# Penis Enlargement by Penile Suspensory Ligament Division with Cross-Plasty of the Skin

**DOI:** 10.5152/tud.2022.21242

**Published:** 2022-03-01

**Authors:** Mykola I. Boiko, Mykola S. Notsek, Oleksandr M. Boiko, Ihor S. Chernokulskyi

**Affiliations:** 1 Department of State Administrative, State Institution of Science, Research and Practical Center of Preventive and Clinical Medicine, State Administrative Department, Kyiv, Ukraine; 2 Clinic “Androcentr”, Kyiv, Ukraine; 3 Primary Care, Cantabria Health Service, Santander, Spain

**Keywords:** penile enhancement, penile enlargement, penis lengthening, ligamentolysis, penile dysmorphic disorder, cross-method

## Abstract

**Objective:**

Current methods of surgical lengthening do not always produce good results and have certain disadvantages. Thus, we conducted this study to demonstrate a modified technique of ligamentolysis that lacks some disadvantages.

**Material and methods:**

We have reviewed 30 patients who underwent surgery with the use of the proposed “cross-method” and also compared with 35 patients who had surgery with the division of the suspensory ligament performed with the use of V-Y plasty method.

**Results:**

We have achieved better enlargement and SEAR (self-esteem and relationship) scores with the “cross-method” compared to V-Y plasty.

**Conclusion:**

The cross-method is a simple alternative technique for penile lengthening that can be performed safely in order to treat small penis syndrome and obtain better results.

**Main Points**

- The suspensory ligament division can be safely performed using the cross-plasty of the skin.
- Penile suspensory ligament division with cross-plasty of the skin gives the better cosmetic result and penile length gain compared to the inverted V-Y technique.
- Penile suspensory ligament division with cross-plasty of the skin leads to increased self-esteem and much greater satisfaction of the patients’ sexual life.

## Introduction

In primitive society, physical characteristics such as body size, strength, and fertility (symbolized by the penis) divided dominant individuals into clans. Extraordinary social and psychological properties were put into the penis. The large and well-functioning male genitals were associated with courage and masculine power, which gave rise to the cult of phallus.^1,2^

Views and beliefs have been changed over the time, but the strive to be the leader is the same. Therefore, the topic of augmentational phaloplasty does not lose its relevance today. The solution of this question lies at the intersection of such specialties as urology, andrology, psychology and plastic surgery, which not only generates a lot of discussions in the scientific literature but also stimulates appearance of new effective surgical techniques.^3^

Nowadays, men often feel the need to enlarge their penis to improve self-esteem, satisfy and impress their partners, and look better than others (locker room syndrome). Thus, it is much more common that men with normal-sized genitals seeking for penis enlargement, than men with small ones.^4,5^ This dissatisfaction is called the small penis syndrome (SPS). Such men do not suffer from severe discomfort in daily and sexual life. They also have normal libido but experience lower sexual satisfaction during the sexual activity.^5^ Furthermore, as we know from literature, men with SPS get more sexual satisfaction after penis enlargement.^6,7^

SPS should be distinguished from Penile Dysmorphic Disorder (PDD), since in both states men are dissatisfied with the penile size.^6,8^ PDD is related to Body Dysmorphic Disorder (BDD), according DSM-5.^9^ The key difference is that BDD causes significant disturbances in various spheres of living. Thus, if SPS presents as a preoccupation with the penis size for at least 1 hour per day, often with repetitive behaviors, such as checking and significant distress or impairment, this is defined as BDD.^10^ Unlike men with SPS, patients with PDD usually are not satisfied with the phallus enlargement. The cases of worsening symptoms were also reported.^6,7^

In recent years, penis enlargement operations have become more popular. The surgical methods, along with the nonsurgical ones, become more common, especially in private institutions.^6^ However, this procedure is still not standardized, leading to a variety of procedures with inconclusive and poorly documented results.^11^ Current methods of surgical lengthening do not always produce good results and have certain disadvantages. At present, the method of division of the penile suspensory ligament (ligamentolysis), in combination with V-Y-shaped skin plasty, is most widely used to enlarge the penis. Its disadvantages are the high probability of scar deformation and the occurrence of penile retraction, insufficient cosmetic effect, and, as a consequence, low patient’s satisfaction with the result of surgery.^3^

Thus, we conducted this study to demonstrate a modified technique of ligamentolysis that lacks some of these disadvantages.

## Material and Methods

This single center, open label study was planned as a randomized controlled trial. it was calculated that 65 people would be sufficient to achieve a 5% alpha error and 20% beta error. A computer-based random number sequence generator was used by the researchers for the randomization method (www.random.org). The participants were enrolled in the study by the principal researcher. Because of the feasibility and nature of the study, the principal researcher and participants were not blinded during allocation to groups.

**Figure 1.**
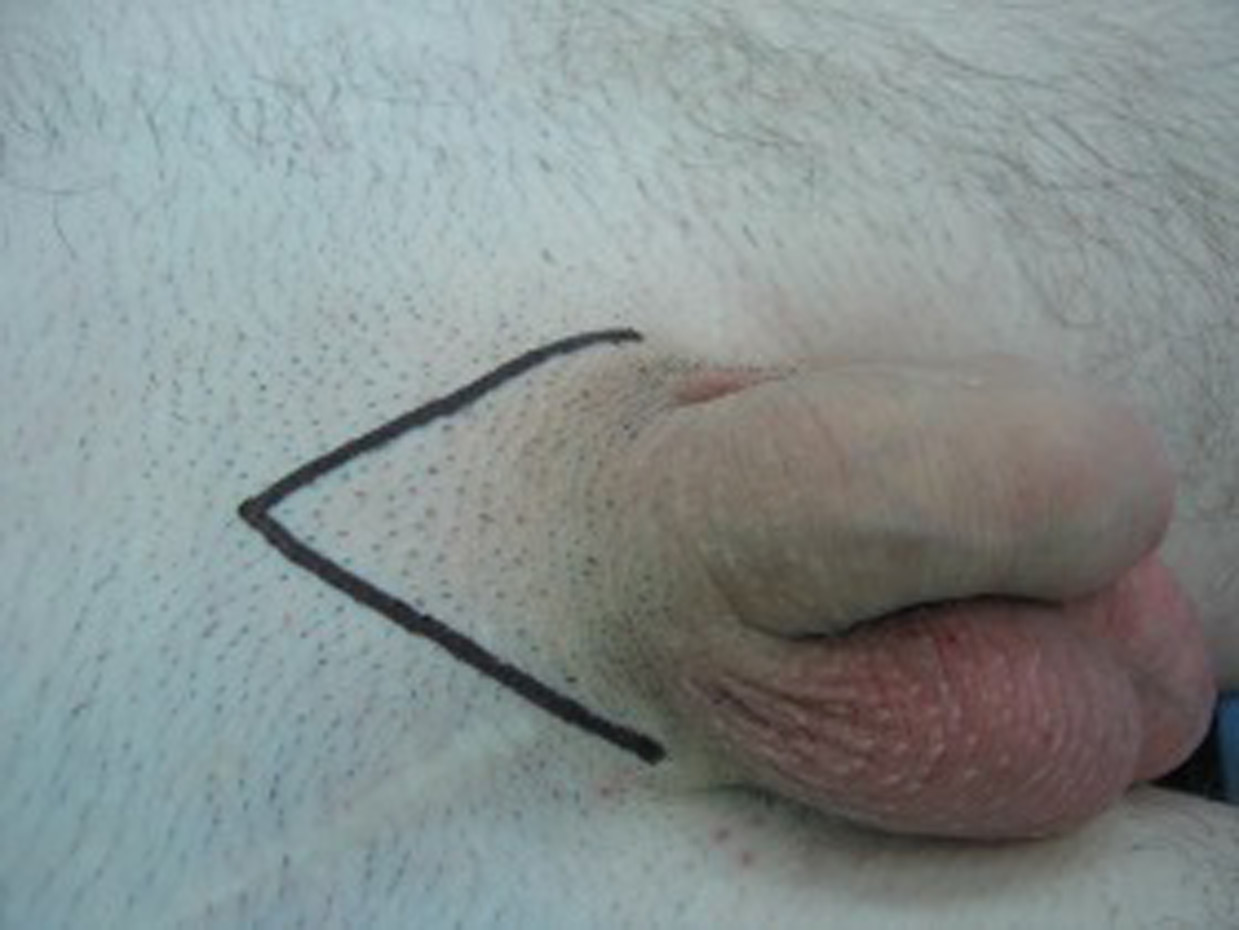
Incision markup for inverted V-Y plasty.

We performed 65 surgeries over the 3-year period (2015–2018). Patients were divided into 2 groups: 35 patients were included in the group where the division of the suspensory ligament was performed by the V-Y plasty (VYG) method (Figure 1), and 30 patients were included in the study group where the surgery was performed using the proposed “cross-method” (CMG). The follow-up period was 3 months.

To evaluate pre- and postoperative self-esteem status of patients, we used “self-esteem and relationship” (SEAR) questionnaire. The questionnaire consists of measurement of sexual relationship satisfaction, overall relationship satisfaction, confidence, and particularly self-esteem in men, where person can get between 14 and 70 points (more points conclude less impairment in above characteristics). The length of the penis in the flaccid state was measured from the penis base to the tip of the glans. The obtained preoperative data are displayed in Table 1.

All men who wished to enlarge their penises underwent a thorough medical examination. Psychosexual, neurological, urogenital, and hormonal aspects of the anamnesis of patients have been studied. Each patient was counselled by the psychologist for PDD. All patients received counselling and reassurance concerning the normal penis size.

All patients had SPS, thus they had a penis of normal size. Micropenis was considered to be any penis with the size that differed by more than 2 standard deviations from the mean or that was less than 7.5 cm in length in the flaccid/erect state.^12^ All patients were warned of their normal size penis and possible complications of surgery, as well as the absence of a direct effect of the elongation surgery on the partner’s sexual satisfaction. All patients were sexually active; 47 of them had regular partnership sexual activity and 18 had irregular one.

Once the relevant information about operation and possible complications were discussed before surgery, all patients signed the corresponding informed consent. All patients consented to the scientific use of their research data without providing any personal data.

Patients with SPS were chosen to the inclusion criteria. Exclusion criteria included patients with psychiatric disorders (such as PDD), central nervous system abnormalities, erectile dysfunction, and patients with micropenis.

The median age of the patients was 32 years. In the study group, this indicator was 32 years. The youngest patient was 18 years old and the oldest one was 58 years old. In the control group, the median age of the patients was 29 years. The youngest patient was 18 years old and the oldest patient was 46 years old.

The length of the penis was measured from the pubo-penile skin junction to the meatus—in accordance with the method proposed by Wessells.^13^ Measurements were made in a flaccid state and at room temperature by the same doctor each time. Patients were calm during the measuring.

**Figure 3.**
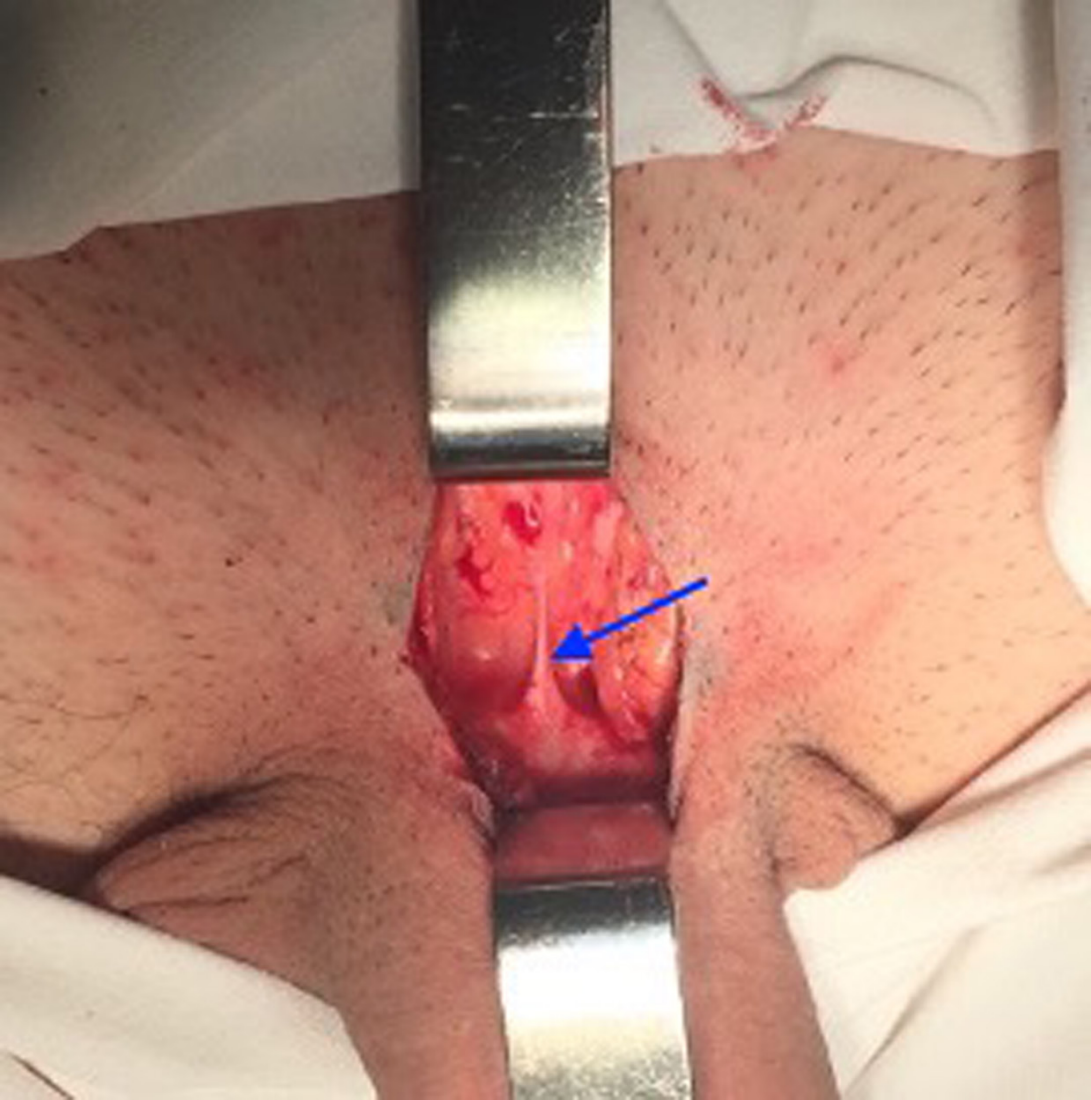
Suspensory ligament of the penis.

*Surgical technique (cross-method). *We begin with 3–4 cm transverse incision of the skin in the area of the penis base, 0.7–1 cm up of the penopubical angle (Figure 2.1, Figure 2.2). Further, suspensory ligament of the penis is located and released (Figure 3).

**Figure 5.**
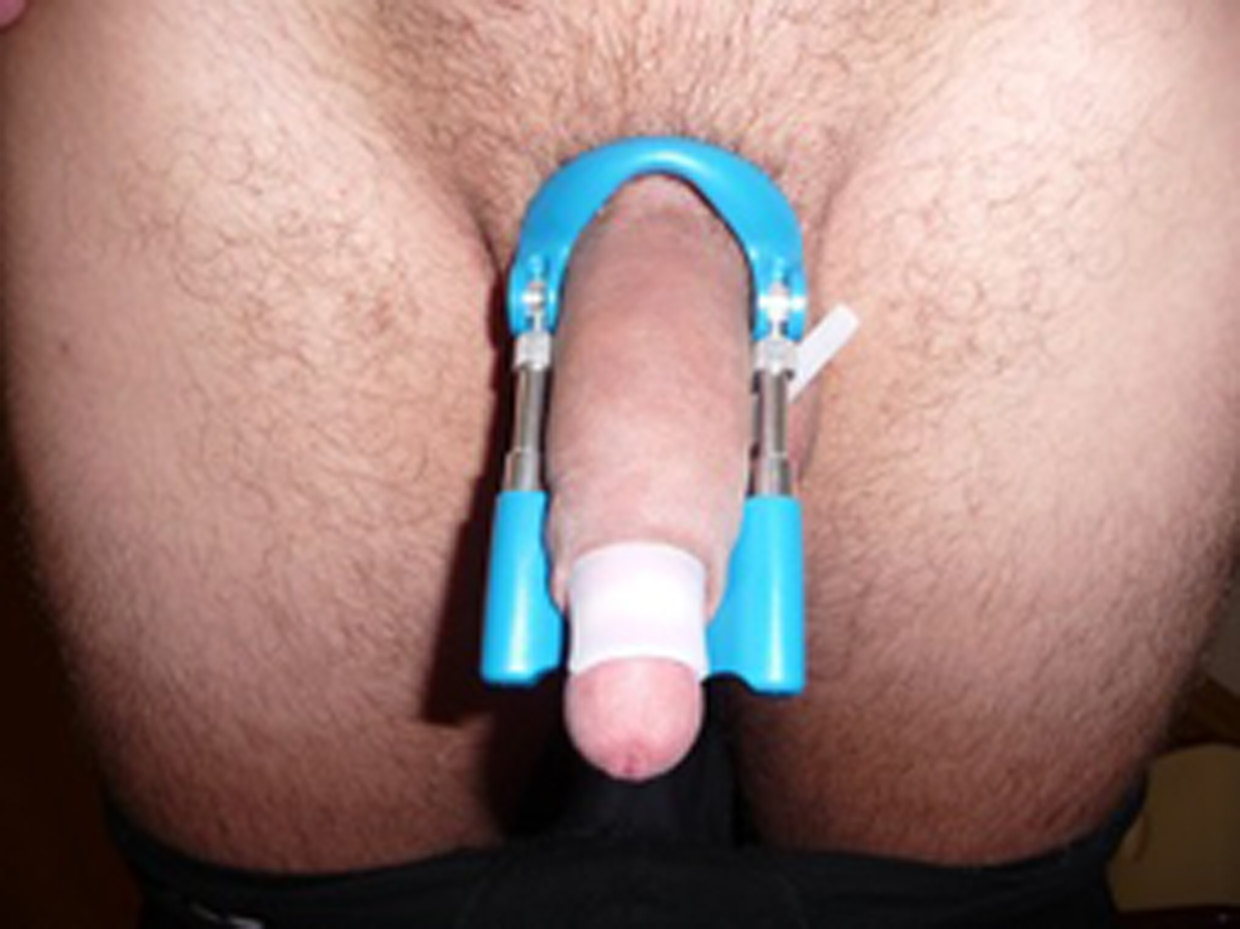
Penile extender.

After completion of the ligamentolysis, the mobilization of the cavernous bodies of the penis from the symphysis is performed to the level of deep arteries entry. An additional dissection of the lateral bundles of the Scarp fascia is performed when the penis is pulled down. As these steps completed, physiological penile curvature disappears on traction and penis is enlarged by 1–3 cm. A spacer (Figure 4.1, Figure 4.2, Figure 4.3) made of certified medical silicone that could be adjusted intraoperatively is fixed to the pubic symphysis, on the place where suspensory ligament was attached, with a non-absorbable suture to prevent the reattachment of cavernous bodies to the pubic symphysis. The cavernous bodies are refixed with a non-absorbable suture to the skin of the penoscrotal angle in the position of its tension (Figure 2.2). The wound is sutured longitudinally (Figure 2.2), thus moving penopubical angle up. All patients were recommended to use a penile extender after wound recovery starting 3 weeks after surgery, for 4–6 hours 2–3 times a week, upto 3–4 months as a preventive measure of reattachment (Figure 5).

Operations were performed under general combined anesthesia. The average duration of surgery in the study group is 107 minutes (87–119 minutes), and in the control group, the average duration is 145 minutes (119–161 minutes).^14-17^ Patients were admitted to the hospital 1 day after surgery. Overall, 7 incidences of complications after surgery were recorded.

*Statistical analysis*. The data of the study were analyzed using the Statistical Package for the Social Sciences (SPSS) version 23 (IBM SPSS Corp.; Armonk, NY, USA). Preoperative and postoperative data were presented as a number, percentage, mean, standard deviation, median, minimum, and maximum. The Shapiro–Wilk test was used to assess suitability for normal distribution. For all quantities that have a normal distribution, a parametric method, the paired sample student’s t-test, was used to assess the differences between the 2 groups. The level statistical significance was set at 5% (*P* < .05).

The manuscript is allowed for publication in open sources by the local Institutional Review Board of State Institution of Science “Research and Practical Center of Preventive and Clinical Medicine,” protocol from November 03, 2020. The study was also approved by the ethics committee of State Institution of Science “Research and Practical Center of Preventive and Clinical Medicine” (protocol №02 from 05.02.2020).

## Results

Totally, 65 suspensory ligament divisions were performed. The preoperative characteristics of 2 groups were similar (Table 1). The mean length of the flaccid penis was 7.6 ± 0.93 cm (5.9–9.3 cm) in the VYG and 7.8 ± 0.94 cm (5.9–10.1 cm) in the CMG.

**Figure 6.**
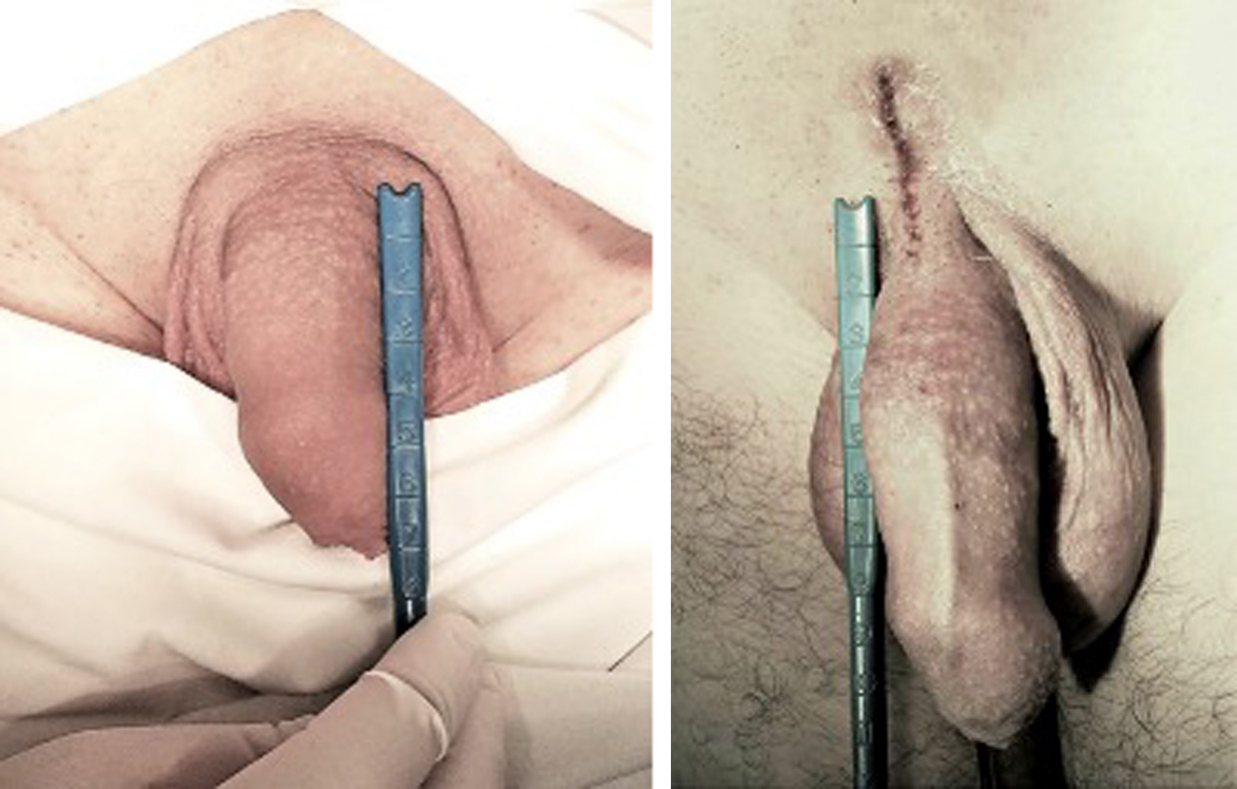
Before and after suspensory ligament division using the cross-method.

In the VYG, the mean increase in length after surgery was 1.6 ± 0.17 cm, and in the CMG (Figure 6) was 2.8 ± 0.31 cm (Table 2). For both groups, the values are statistically significant (*P* < .001). There was also a significant increase in mean enlargement in the CMG comparing to the VYG 1.2 ± 0.4 cm (*P* < .001) Table 3).

Regarding SEAR questionnaire, the satisfaction of the sexual life improved in each group, compared with the results before surgery: mean 7.6 ± 2.53 (*P* < .001) points in the CMG and mean 5.8 ± 1.39 (*P* < .001) in the VYG (Table 2). If one compares these values, it is evident that the mean results of the questionnaire in the CMG are 1.8 ± 3.13 points higher than in the VYG (*P* = .004) (Table 3), which means there was an improvement in SEAR satisfaction in both groups, but in CMG it is more prominent.

No difficulties in sexual activity or functional problems were reported in the postoperative period. However, minor complications were documented. We registered 4 incidences (11%) of hypertrophic scars in the VYG. In CMG, there were 3 incidences (10%): 2 (7%) patients with hypertrophic scars and 1 patient (3%) with the marginal wound dehiscence. No incidences of infection and postoperative bleeding were detected.

## Discussion

For the first time, the data on the normal length of the penis were published in 1899 by H. Loeb, according to which the average length of the flaccid penis was 9.5 cm. According to most authors, the average (normal) length of the penis in the erect state is in the range of 12–18 cm and the circumference is 9.5–11.5 cm. In the flaccid state, the normal length is 7.5–10 cm and the circumference is about 7–9 cm.^18^

Usually, augmentational phalloplasty is devoted to pathological states such as micropenis and hidden penis. But in the vast majority of cases, it is performed at the normal size penis for aesthetic purposes, with diagnosis of PDD, SPS, low self-esteem, and insecurity of the man.^4^

Division of penile suspensory ligament or ligamentolysis is a simple and commonly used technique for penile lengthening. Some sources document serious morbidity rate related to this procedure,^19,20^ on the other hand, other studies showed the low complication rate.^21^ Ligamentolysis is quite a fast and simple method that gives results similar to other more complex penile lengthening procedures.^22^ In fact, the absolute length of penis does not change. During the surgery only visible external part of penis is made longer.

The newest studies of the penile suspensory ligament division techniques report a variety of results ranging from 1 cm up to 5.1 cm increase that could be explained by different approaches to measurement. As reported by Protogerou et al.^23^ a 5.1 cm increase in penile length after the enlargement surgery was achieved. In our study, postoperative increase in penis length (1.6 cm and 2.8 cm) can be compared to the available reviews of the V-Y plasty method. In the review by Vardi Y et al.^3^ the average increase in length of 1–2 cm is indicated.^4,11,12^

Postsurgical complications such as hypertrophic scars, hair-bearing skin flap, infections, nodal formations, and penile deformations are the most serious complications of penile lengthening surgeries.^19,20^ Some of them were observed in 7 patients during our research period. This was reflected in SEAR scores in that patients and had some effect on overall statistical results.

Another serious consideration regarding ligamentolysis is postoperative shortening of penis that was described by some scientists.^3,11,24^ It is stated that such complication is a result of fibrous tissues formation in the place of ligament division that reattach penile shaft to the pubis. But the method to avoid reattachment is also known. The spacer placement between the penis and the pubis prevents possible shortening.^25,26^ This technique was used in our research in both groups of patients and, as a result, no cases of penile shortening in the postoperative period was observed. There were no cases of dorsal nerve injury and osteitis, nor silicone spacer infection. Cases of spacer malpositioning and complications leading to spacer removal were not observed in both groups. Also, such complications are not described in the literature.^26,27^

Moreover, the penile traction device (extender) is a preferable option to improve the result of surgery as it is affordable and easy to maintain and setup and also gives positive outcomes.^27,28^

Thus, this procedure doesn’t ensure total cure for PDD because normal penile size is always normal in all circumstances and the procedure can only diminish patients’ anxiety.^26^ The self-esteem of patients remarkably increases after surgery that positively affect their life quality. The significant improvement of satisfaction and self-esteem scores demonstrates this statement.

**Figure 7.**
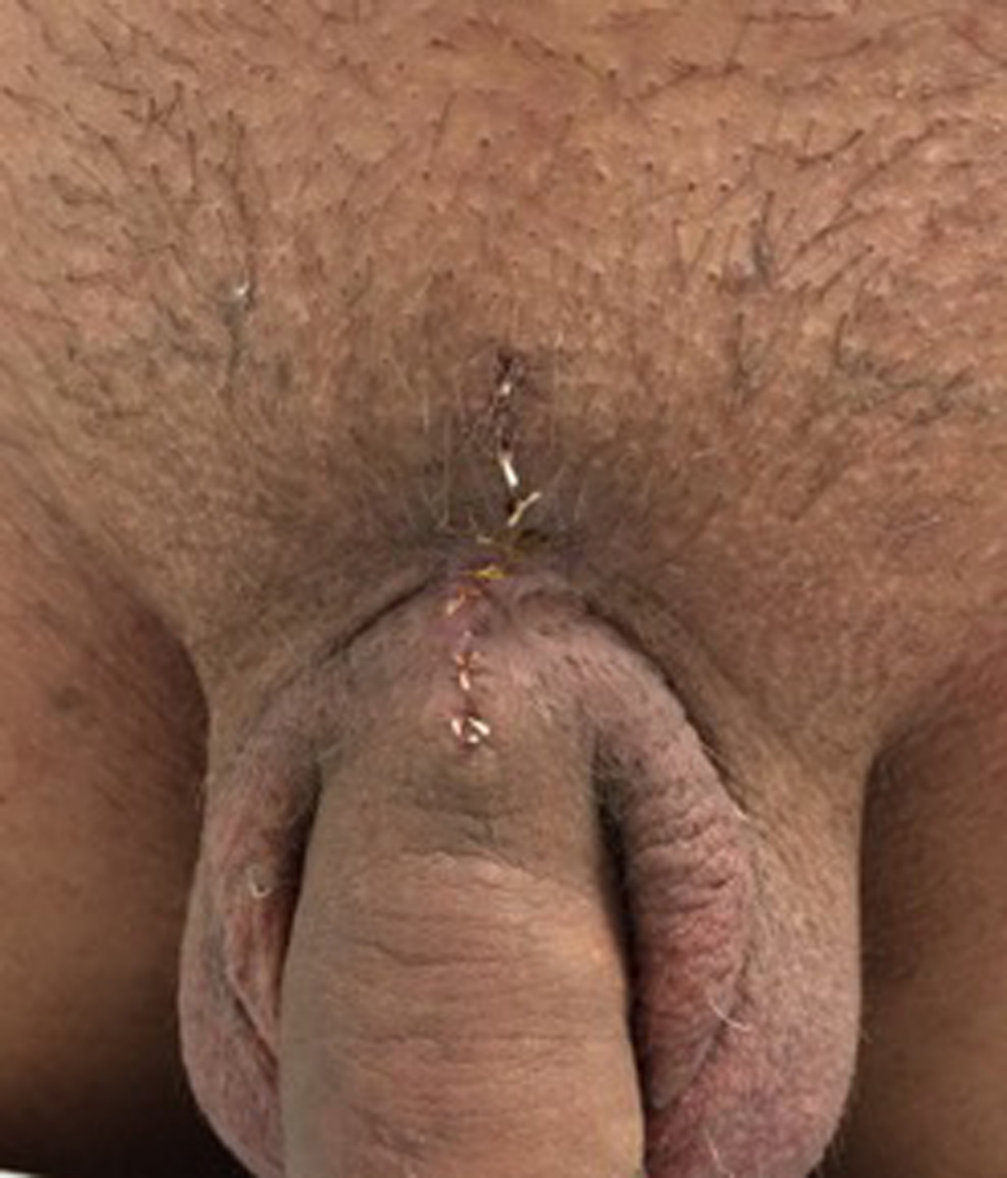
The view after surgery performed using the proposed cross-method.

The other part that positively affects self-esteem and postoperative satisfaction is aesthetics.^27^ The division of the penile suspensory ligament can be performed through a simple transverse incision or some more complicated ones in order to avoid scar contracture and shortening of the length. For this purpose, several skin plasty methods were investigated in the literature: M-plasty, V-Y-plasty (the most common and widely used), Z-plasty, and double Z-plasty.^28–30^ In our opinion, cross-method (Figure 7) has much more preferable cosmetic results compared to other methods and good outcomes regarding the scar contracture formation. Furthermore, overall scar length after cross-method is shorter, thus less visible in postoperative period. There is a similar option of skin plasty that starts with transverse incision proposed by Monreal J^28^ transforming into Y-formed plasty during the time of suturing.

The limitations of our study are the small study groups and short-term follow-up.

In conclusion, the suspensory ligament division (ligamentolysis) can be safely performed using the cross-plasty of the skin. The proposed method leads to increased self-esteem and much greater satisfaction of the patients’ sexual life. Our cross-technique can be recommended as the preferred method of augmentation phalloplasty in other centers for patients with the penile dysmorphophobia.

### Declaration of Interests

The authors have no conflicts of interest to declare.

## Figures and Tables

**Table 1.** Investigated Data in Groups Before Augmentative Phalloplasty (*P* < .05)

Measurements	VYG	CMG
Number	35	30
Age	29 (18–46)	32 (18–58)
Penile length (cm)	7.6 ± 0.93	7.8 ± 0.94
SEAR scores	31.8 ± 4.19	32 ± 5.26

CMG, cross-method; VYG, V-Y plasty; SEAR, self-esteem and relationship.

**Figure 2.**
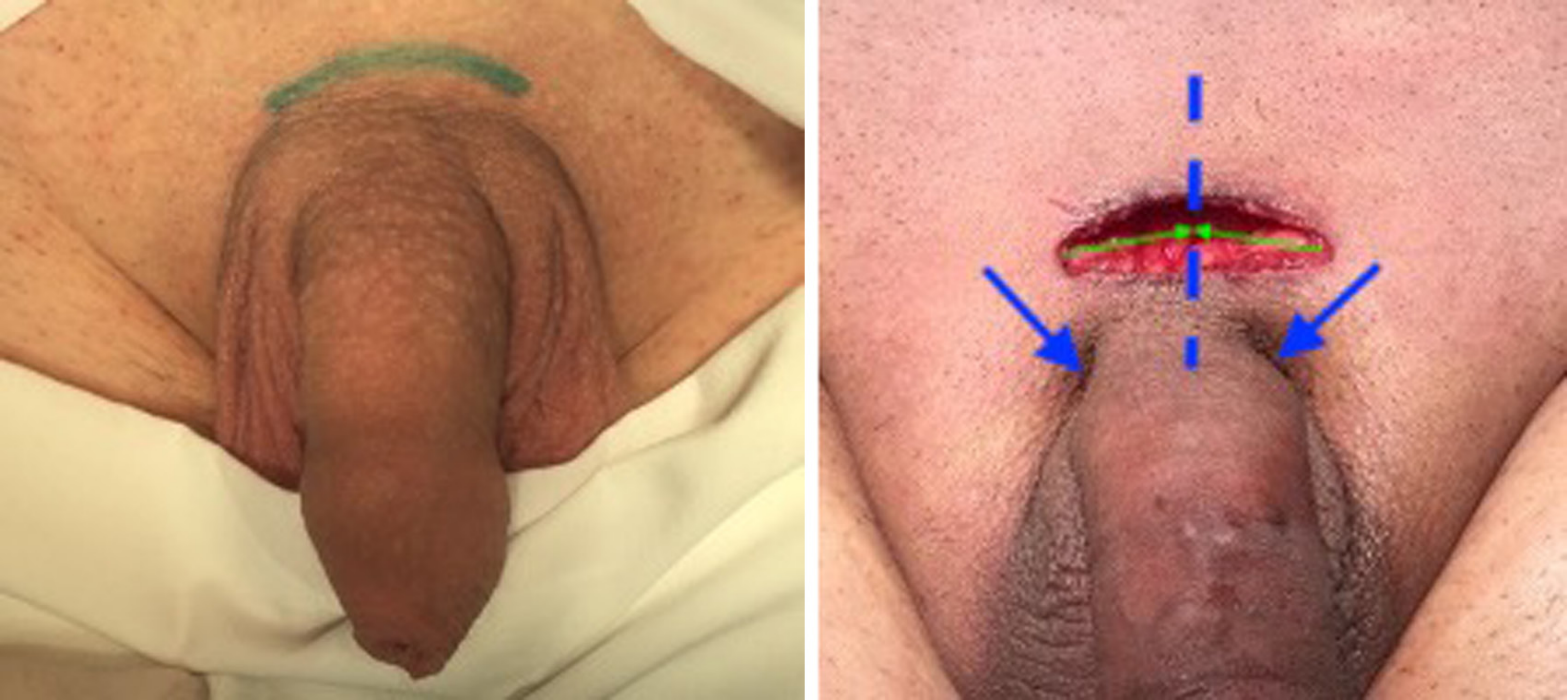
(1) Markup according to the developed cross-method; (2) Incision with schematic markup of the suture line (blue dash line) and refixation dots of cavernous bodies to the skin (marked with blue arrows).

**Figure 4.**
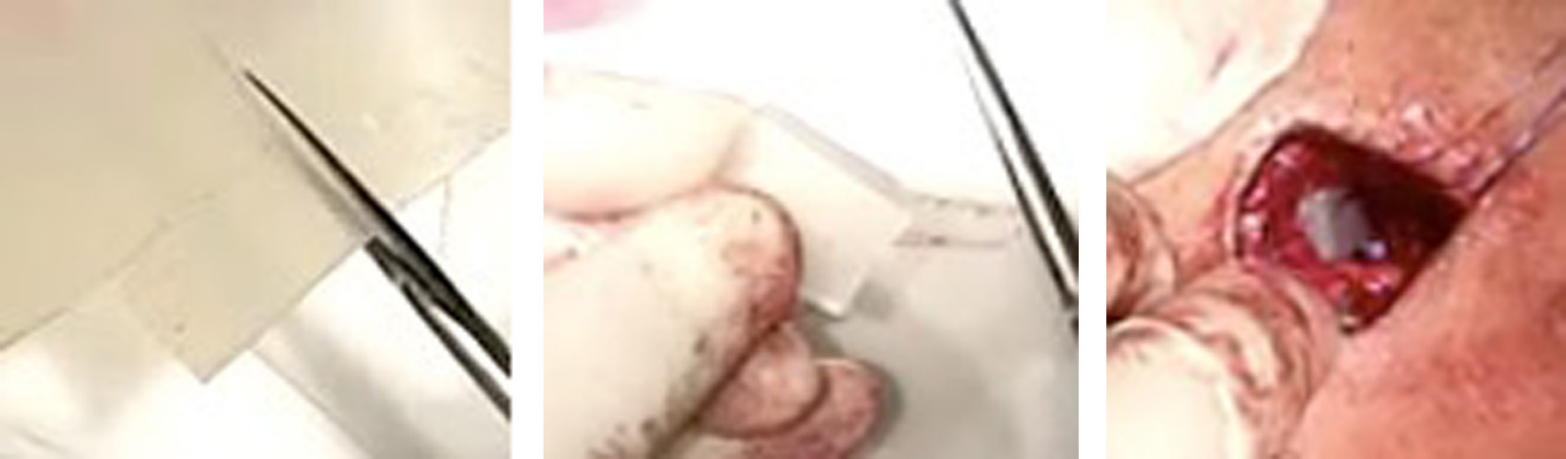
(1) Adjustment of silicone spacer; (2) Silicone spacer before insertion; (3) Inserted silicone spacer.

**Table 2.** Changes in the Observed Values After Surgery

Mean values	VYG	CMG
Penis enlargement after surgery (cm) (*Р* < .001)	1.6 ± 0.17	2.8 ± 0.31
Change of SEAR score (*Р* < .001)	5.8 ± 1.39	7.6 ± 2.53

CMG, cross-method; VYG, V-Y plasty; SEAR, self-esteem and relationship.

**Table 3.** Comparison of Results in CMG with VYG

Groups Comparison	Mean Increase	*P* (Two-Tailed Student’s t-Test)
Enlargement (cm)	1.2 ± 0.4	<.001
Rise of SEAR score	1.8 ± 3.13	.004

CMG, cross-method; VYG, V-Y plasty; SEAR, self-esteem and relationship.
